# Urine-derived stem cells: a sustainable resource for advancing personalized medicine and dental regeneration

**DOI:** 10.3389/fbioe.2025.1571066

**Published:** 2025-04-28

**Authors:** Gamal A. Atia, Ahmed Abdal Dayem, Ehab S. Taher, Wafaa Y. Alghonemy, Ssang-Goo Cho, Ahmed A. Aldarmahi, Md Azizul Haque, Abeer Alshambky, Noha Taymour, Ateya M. Ibrahim, Donia E. Zaghamir, Ekramy M. Elmorsy, Helal F. Hetta, Mohamed E. Mohamed, Kasim S. Abass, Shifan Khanday, Ahmed Abdeen

**Affiliations:** ^1^ Department of Oral Medicine, Periodontology, and Diagnosis, Faculty of Dentistry, Suez Canal University, Ismailia, Egypt; ^2^ Department of Stem Cell and Regenerative Biotechnology, School of Advanced Biotechnology, Molecular & Cellular Reprogramming Center, Institute of Advanced Regenerative Science, and Institute of Health, Aging & Society, Konkuk University, Seoul, Republic of Korea; ^3^ Department of Basic and Clinical Medical Sciences, Faculty of Dentistry, Zarqa University, Zarqa, Jordan; ^4^ R&D Team, StemExOne Co., Ltd., Seoul, Republic of Korea; ^5^ Department of Basic Science, College of Science and Health Professions, King Saud bin Abdulaziz University for Health Sciences, Jeddah, Saudi Arabia; ^6^ National Guard- Health Affairs, King Abdullah International Medical Research Centre, Jeddah, Saudi Arabia; ^7^ Department of Biotechnology, Yeungnam University, Gyeongsan, Republic of Korea; ^8^ Molecular Therapeutics Program, Fox Chase Cancer Center, Temple University, Philadelphia, PA, United States; ^9^ Department of Biochemistry, Animal Health Research Institute, Cairo, Egypt; ^10^ Department of Substitutive Dental Sciences, College of Dentistry, Imam Abdulrahman Bin Faisal University, Dammam, Saudi Arabia; ^11^ College of Nursing, Prince Sattam bin Abdulaziz University, Al-Kharj, Saudi Arabia; ^12^ Center for Health Research, Northern Border University, Arar, Saudi Arabia; ^13^ Department of Natural Products and Alternative Medicine, Faculty of Pharmacy, University of Tabuk, Tabuk, Saudi Arabia; ^14^ Department of Basic Medical Sciences, College of Medicine, AlMaarefa University, Riyadh, Saudi Arabia; ^15^ Department of Physiology, Biochemistry, and Pharmacology, College of Veterinary Medicine, University of Kirkuk, Kirkuk, Iraq; ^16^ Department of Biomedical Sciences, Dubai Medical College for Girls, Dubai Medical University, Dubai, United Arab Emirates; ^17^ Department of Forensic Medicine and Toxicology, Faculty of Veterinary Medicine, Benha University, Toukh, Egypt

**Keywords:** regenerative medicine, non‐invasive cell therapy, disease modeling, cartilage repair, exosomes, biomaterials, tissue engineering

## Abstract

Urine-based therapy, an ancient practice, has been utilized across numerous civilizations to address a wide range of ailments. Urine was considered a priceless resource in numerous traditional therapeutic applications due to its reported medicinal capabilities. While the utilization of urine treatment is contentious and lacks significant support from modern healthcare, the discovery of urine-derived stem cells (UDSCs) has introduced a promising avenue for cell-based therapy. UDSCs offer a noninvasive and easily repeatable collection method, making them a practical and viable option for therapeutic applications. Research has shown that UDSCs contribute to organ preservation by promoting revascularization and decreasing inflammatory reactions in many diseases and conditions. This review will outline the contemporary status of UDSCs research and explore their potential applications in both fundamental science and medical practice.

## Graphical Abstract

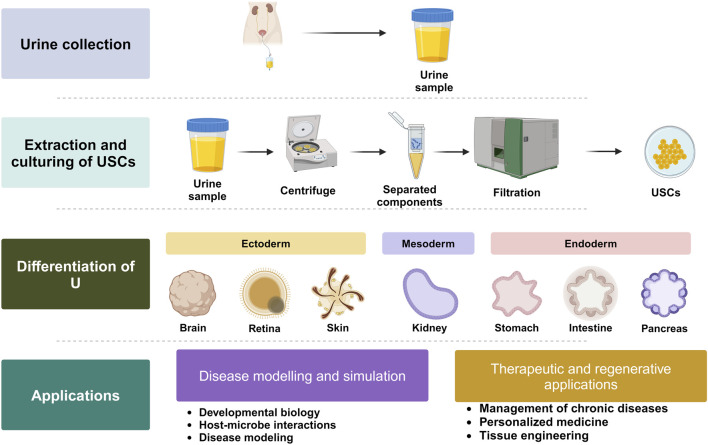

## 1 Introduction

Regenerative medicine can restore damaged or incapacitated organs. Stem cells have made significant progress and are an increasingly popular approach in regenerative applications thanks to their tremendous multiplying ability and multi-differentiation potential (Augustine et al., 2021). Since all tissues include endogenous stem cells that are crucial for their equilibrium, they are usually recognized as a more accessible cell reservoir for use in therapy and remain an expanding and fascinating field with the promise to improve human healthcare (Liu et al., 2020a). Urine-derived stem cells (UDSCs) have developed as a potential cellular origin in the last decade due to their noninvasive acquiring process, robust proliferation capacity, and diverse medical applications and act as the starting point in order to convert them into disease-tailored induced pluripotent stem cells (IPSCs). UDSCs are now playing a significant part in adult stem cell biology (Barakat et al., 2020; Ji et al., 2017).

Sutherland and Bain announced the practical separation of live cells from urine utilizing specimens of newborns (Sutherland and Bain, 1972). Several teams repeated the process in subsequent years, and cells were taken from individuals with various illnesses. These urinary cells had a variety of topologies (polygonal or elongated), but the source was primarily epithelial (from the kidney tubules and urothelial) according to biomarker gene transcription (Kibschull et al., 2023; Zhuang et al., 2021; Sun et al., 2022b; Lone et al., 2022).

## 2 Extraction of UDSCs

As previously mentioned, Sutherland and Bain published the first research to describe the gathering of exfoliated urine cells in 1972. They obtained proliferating cells from the urine of four newborns under the age of 2 days (Sutherland and Bain, 1972). Zhang et al. identified renal progenitors in cultivated urine-derived cells from 15 healthy persons and eight patients with vesicoureteral reflux. There were around 2–7 progenitor-like cells per 100 mL of urine, capable of forming a homogeneous, concentrated colony from an individual cell in 2 weeks. These cells may develop *in vitro* for eight passages before differentiating into urothelial, smooth muscle, endothelial, and interstitial cells. Nevertheless, the fraction of cells that display stem/progenitor characteristics declined after every passage (Zhang et al., 2008).

Following gathering urine, it should be centrifuged to extract UDSCs, and then contaminated cells should be progressively eliminated. Urine cryopreservation from healthy young individuals is the most effective sample for extracting fresh UDSCs from samples taken from diseased persons, especially those suffering from diabetes (Zhao et al., 2018).

However, the most recent research demonstrates that they can be effectively obtained from these patients, but their regenerating capacity has been substantially decreased, making them unsuitable for treatment (Ouyang et al., 2019). After collecting the appropriate specimens, an antibiotic is applied to minimize contamination. After centrifuging, the remaining residue is eliminated, followed by two washes with phosphate buffer saline (PBS) and resuspension of the material. Because urine involves many types of cells, it is vital for identifying the different contents following extraction (Kim et al., 2020).

## 3 UDSCs culture

Separation of UDSC from fresh urine is more appropriate, but procedures for UDSC isolation could be accomplished within 24 h if urine is kept at 4°C in a storage medium with serum; prolonged preservation negatively affects the lifespan of cells (Lang et al., 2013). Investigations on urine storage for UDSCs separation are still limited, and it is a crucial subject to tackle as it would provide numerous benefits to the methodology of UDSCs separation (Zeng et al., 2022; Xie et al., 2023). Bharadwaj et al. reported an average of 3.7 × 108 UDSC cells at passage five after 27 days of culture. Nevertheless, amounts may vary depending on conditions (Bharadwaj et al., 2011). UDSCs may be separate from persons of both sexes, and the age range documented so far is 5–75 years (Kim et al., 2023). Telomerase activity in adult cells is mainly restricted to stem cells and is linked with strong proliferating ability. Bharadwaj et al. reported telomerase activity in 60% of the separate UDSC specimens (Bharadwaj et al., 2013), whereas standard genome sequencing was discovered at least until cell passage 15 (Kibschull et al., 2023).

Moreover, Chun et al. suggested implementing a 5% O2 hypoxic medium with collagen type I as a tailored technique for expanding collected UDSCs while preserving their chromosomal integrity and multipotent development capacity (Chun et al., 2016).

A new investigation found that flavonoids enriched with Matrigel. Flavonoids might improve UDSCs isolation, yield, colony-building capability, and differentiation capability (Kim et al., 2020). Nevertheless, to create a significant amount of developed cells before therapeutic administration, maximal growth of UDSCs is frequently necessary *in vitro*, resulting in cellular aging following several passages (Shi et al., 2022).

Liu et al. used undifferentiated UDSCs throughout the initial phases of *in vitro* passaging (≤p3) with a heparin hyaluronic-acid hydrogel (hp-HA) gel encapsulating growth factors. Following subcutaneous insertion *in vivo*, the cells demonstrated enhanced survival, integration, vascular, neuronal, and myogenic transformation capabilities (Liu et al., 2020b).

## 4 Properties of HUDSCs

**FIGURE 1:**
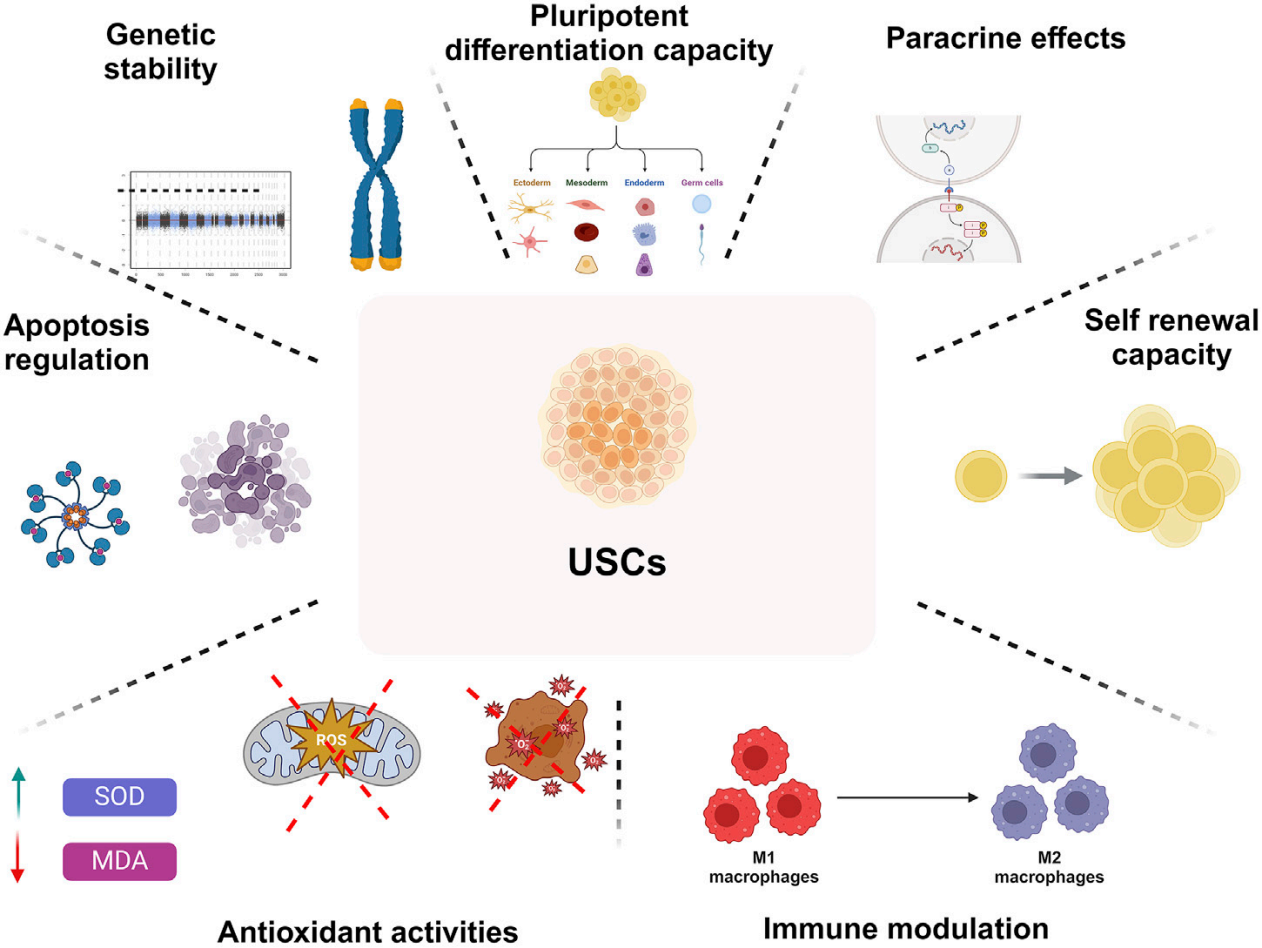
An overview on biological characteristics of UDSCs regrading pluripotent differentiation capacity, paracrine effects, self-renewing capacity, immune-modulating characteristics, antioxidant activities, modulation of apoptosis, and genetic stability.

Urine contains multiple cell types, generically categorized as UDSCs, containing numerous cellular groups and stem cells. UDSCs have MSC-like characteristics and possess the capacity to divide and multiply into diverse cell lines. The essential sources and particular mechanisms that underpin the formation and evacuation of UDSCs are yet unknown. In addition, they may be lost in urine due to the normal cycle of homeostatic tissue repair and renewal. Urine stem cells are proven to have several outstanding biological features (Figure 1) (Zhou et al., 2012).

### 4.1 Genomic durability

*In vitro* multiplication of UDSCs is required to generate a substantial number of cells for therapeutics because of their small quantity in human urine; however, this method enhances the danger of genetic changes and cellular mutations. Genetic integrity is an important safety issue for stem cell-based therapies since destabilization in the genome, including chromosomal changes, is linked to higher tumor formation (Wu et al., 2011). Telomere degradation during the proliferation of cells leads to cell death and mortality. Telomere length is critical for the continued or limitless multiplication of self-renewing cells. Cells’ telomerase transcription (low or undetectable) should be evaluated to exclude the potential of tumorigenicity. UDSCs’ powerful dividing capability is linked to their lengthy telomeres. Surprisingly, specific UDSCs clones exhibited apparent amounts of telomerase activity during the initial stages, and their dividing capacity was greater than that of telomerase-negative cells (Shi et al., 2022). Nonetheless, telomerase activity gradually dropped throughout cellular subculture. As a result, notwithstanding identifiable telomerase activity within specific UDSCs clones, *in vivo* investigations show that USCs did not become tumorigenic upon growth.

### 4.2 Differentiation capacity

**FIGURE 2:**
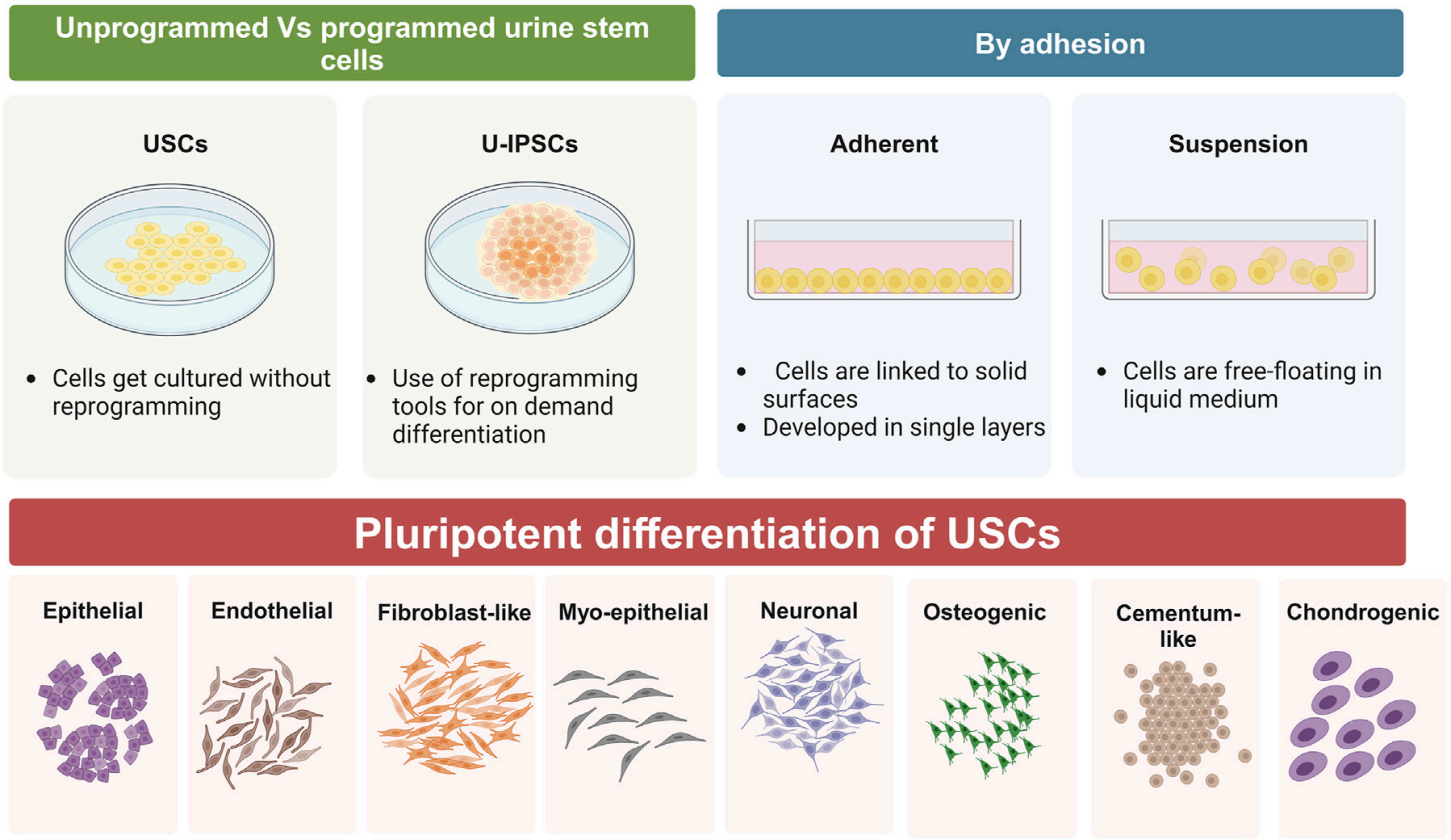
Schematic representation of differentiation capacity of UDSCs, whether they were used as programmed or unprogrammed cells, they have an outstanding tendency to form epithelial, endothelial, fibroblast-like, myo-epithelial, neurons-like, osteoblasts, cementoblasts, and chondrocytes.

Autologous somatic stem cells provide a unique benefit for upcoming therapeutic uses since they seldom cause immunological rejection (Jha et al., 2024; Selvido et al., 2023). Furthermore, the capacity to grow rapidly and be driven into multiple cell lineages provides a foundation for stem cell therapies. UDSCs may be grown to produce a vast population, and their adaptability has been extensively established via research (Figure 2; Table 1) (Shi et al., 2016). Initially, UDSCs were cultivated from newborn infants and showed low proliferation capacity (Sutherland and Bain, 1972). In 2008, urological tissue regeneration researchers effectively generated urine cells with remarkable division capacity (Zhang et al., 2008). Once extracted, UDSCs may be grown *in vitro* and divided into various cellular kinds by inducing lineage-specific differentiation under the right circumstances. UDSCs enhance PDLSC division, osteogenic and cementogenic transformation, and regrowth of fresh tissues *in vivo* in a ratio-dependent fashion via noncontact coculture. These findings point to its potential application as a unique strategy for clinical periodontal regeneration (Yang et al., 2020). Human urine-induced pluripotent stem cells (hU-iPSCs)-derived epithelium layers developed into dental-like frameworks, exhibiting physical qualities similar to those exhibited by regular human teeth (Cai et al., 2013).

**TABLE 1:** Differentiation capacities of UDSCs/U-IPSCs.

Cell line	Expressed biomarkers	Reprogramming technique	References
Osteoblasts	CD73, CD90, and CD105		Park et al. (2023)
Cardiomyocytes	Troponin-T	Lentiviruses	Jiang et al. (2018)
	c-Myc and Klf4	Lentiviruse	Guan et al. (2014b)
	Oct4/Sox2, c-Myc, and Klf4	Sendai virus	Cao et al. (2018)
	*NKX2-5, GJA1*, *GJA5* and *RYR2*	Episomal vectors	Jouni et al. (2015)
Hepatocytes	ALB, CYP450, AFP		Zhou et al. (2020)
	CD24, CD29, CD73, CD90, and CD146		Zhang et al. (2021)
	CD24, CD29, andCD146		Hu et al. (2020)
Neural cells	PAX6, SOX1, SOX2 and nestin	Episomal vectors	Wang et al. (2013)
	Sox2- and Nestin	Lentivirus	Guan et al. (2014a)
	MNP, Olig2 and Pax6	Sendai virus	Yi et al. (2018)
Renal cells	PAX2, WT1, and CADHERIN 6		Choi et al. (2017)
Skeletal myocytes	myf5, myoD, myosin, and desmin		Chen et al. (2017)
	PAX7, MYOD, MYOG, and MF20	Episomal vectors	Kibschull et al. (2023)
Chondrocytes	Agg, Col I, Sox9, and Col II		Sun et al. (2021b)
Pancreatic β-cells	Ngn-3 and Pdx-1		Hwang et al. (2019)
Endothelial	CD31, vWF, eNOS		Liu et al. (2018)
Epithelial	FOXA2 and SOX17	Episomal plasmids	Wang et al. (2016)
Cementogenic	CEMP1		Yang et al. (2020)

AFP, Alpha-fetoprotein; Agg, Aggrecans; ALB, Albumin; CADHERIN, calcium-dependent adhesion; CD, Clusters of differentiation; CEMP1, Cementoblastoma-derivedprotein1; c-Myc, Cellular myelocytomatosis oncogene; Col I, Collagen; CYP, Cytochromes-P; FOX, Forkheadbox protein; GJA, Gap juxtaglomerular apparatus; Klf4, Krüppel-like factor 4; MF, Myosin Heavy Chain Antibody; MNP, Manganese peroxidase; Myf, Myogenic factor; MyoD, Myoblast determination protein; Ngn, Neurogenin; Olig, Oligodendrocyte lineage transcription factor; PAX6, Paired-box; PDX, Pancreatic and duodenal homeobox; RYR2, Ryanodine receptor; Sox, Sex determining region Y-box; WT, Wilms tumor.

When stimulated with proper media, UDSCs produce urothelial-specialized biomarkers, including uroplakin-III and cytokeratin. During the differentiation process, cell morphology changed dramatically, resulting in a cuboidal form (Wan et al., 2018). When grown in a myogenic media, UDSCs display muscular-associated biomarkers like myosin (Liu et al., 2013). Intradermal applications in nude mice and USCs with myogenic transformation could create an assortment of smooth muscle cells (Chen et al., 2017).

UDSCs can successfully transform into functional endothelial cells when incubated in an endothelial stimulating medium, exhibiting a cobblestone-like form, expressing endothelial biomarkers, forming tubules on Matrigel, and developing tight connections, analogous to natural endothelial cells (Liu et al., 2018).

Following osteogenic development, UDSCs can generate mineralized matrices. During osteogenic cell commitment, alkaline phosphatase activity and osteogenic biomarker transcription increase (Qin et al., 2014). UDSCs can alter shape to become a neuron-specific architecture with distinct neurogenic extensions (Wu et al., 2020). Additionally, the generated UDSCs exhibit neuronal biomarkers, including Nestin, and Sox2 (Sato et al., 2019).

### 4.3 Paracrine effects

UDSCs have substantial paracrine impacts and could discharge various paracrine substances, including regulatory molecules and exosomes. The UDSCs secretory system has sparked considerable curiosity in tissue repair (Xiang et al., 2020). Human USCs were proven to decrease inflammatory reactions and fibrosis via paracrine mechanisms. When cultivated alongside PDLSCs, UDSCs can promote the osteogenic development of PDLSCs via paracrine mechanisms. Transfected genes have been used to modulate UDSCs synthesis. UDSCs could express the VEGF gene, showing more enhanced angiogenesis. Transplanting UDSCs engineered with the FGF2 gene significantly restored vascularity via paracrine actions in rats. As a crucial paracrine mediator, extracellular vesicles (EVs) carry numerous bioactive cargos (Zidan et al., 2021). Investigations on animals have highlighted the excellent prospects of UDSC-derived EVs (UDSC-EVs). For example, in ischemic mice, USC-EVs significantly improved angiogenesis. Furthermore, UDSCs-EVs can improve tissue repair, which could be due to the microRNAs they contain. UDSCs-exosomes (UDSCs-EXOs) can effectively inhibit osteonecrosis by delivering peptides having proangiogenic and antiapoptotic properties (Wan et al., 2022). UDSCs-EVs are capable of treating osteoporosis in mice by delivering functional proteins. UDSCs-EXOs are also helpful in shielding tissues from injuries (Chen et al., 2020).

### 4.4 Self-renewal potential

UDSCs could be obtained from urine, producing significant cellular quantities from just one copy. These cells produce homogeneous kinds of cells and are excessively proliferating since they have more vigorous telomerase activity and more considerable telomere lengths than other forms of MSCs(Supakul et al., 2023). The majority of UDSCs obtained from young adults displayed telomerase activity (TA+) and maintained extended telomeres sizes Telomerase activities, while UDSCs-TA + dropped to 50%–60% of the UDSCs among individuals 50 years old and beyond (Shi et al., 2012). Regardless of multiple passes, TA+ and TA-USCs maintain standard karyotyping in the growing environment. They did not develop teratomas 3 months following implantation (Shi et al., 2012). About 100–140 UDSC clones can be acquired every 24 h from every person (Zhang and Atala, 2022).

### 4.5 Immune-modulating activities

Recent investigations have revealed that UDSCs implantation can minimize localized inflammation and expedite tissue regeneration by encouraging macrophage polarization toward the M2 phenotype (Sun et al., 2021a; Pei et al., 2023). There was no evident immunological rejection or cancer detected in these investigations, indicating that USCs are low immunogenic and safe for xenotransplantation (Cehakova et al., 2023; Liu et al., 2022).

Host exposure to periodontitis controls not only the shift from microbial symbiosis to dysbiosis but also the onset of inflammation and the advancement of irreversible tissue degradation (Darby, 2022). Periodontitis advancement and severity are influenced by host-related factors, including immunoregulatory dysfunction, immunodeficiency, and systemic diseases associated with periodontitis (Hernández et al., 2021). Deficiencies or dysfunction of the host’s defenses lead to a failure to control dysbiotic microbial populations and the associated disease (Deng et al., 2022). Silk fibroin (SF)/nanohydroxyapatite (nHA) composite loaded with less than 0.5% GO were biocompatible and enhanced UDSCs multiplication and bone formation. It could stimulate M2-type differentiation while inhibiting M1-type conversion of macrophages. It displayed the most substantial potential for boosting the M2-type polarization of macrophages and promoting osteoinduction *in vivo* (Sun et al., 2021a).

### 4.6 Antioxidant activity

Oxidative stress is a hazardous mechanism that can cause malignancies, cardiac illness, neurological problems, respiratory diseases, renal disease, chronic inflammation, and premature aging (Deng et al., 2021). Li et al. discovered that UDSCs-EXOs had antioxidant potential since they were able to enhance SOD activity and decrease MDA levels (Li et al., 2020). Comparably, Zhang et al. discovered higher concentrations of SOD-1 in mice treated with UDSCs (Zhang et al., 2020a). Furthermore, UDSCs have been shown to have an antioxidant impact by significantly lowering the levels of oxidative stress-associated markers (Li et al., 2017a).

### 4.7 Apoptosis regulation

Apoptosis is a type of planned cell mortality that occurs when separate apoptotic bodies arise to preserve equilibrium and manage the normal cell cycle. However, severe apoptosis might degrade tissue function further, leading to serious diseases. As a result, blocking apoptosis is viewed as saving damaged tissue (Xu et al., 2019).

Sun et al. discovered that UDSCs inhibited the formation of Bax and Caspase three while increasing the production of the antiapoptotic protein Bcl-2 (Sun et al., 2019). *An in vivo* study proved that therapy with UDSCs decreased apoptotic levels by significantly lowering caspase-3 and Bax levels while significantly boosting Bcl-2 levels, resulting in fewer apoptosis nuclei (Li et al., 2017a).

TIMP1 is a naturally occurring matrix metalloproteinase (MMP) inhibitor. The PI3K and JNK signaling mechanisms were activated by the recombinant human TIMP1 protein, which prevented apoptosis. TIMP1 was discovered to be exceptionally abundant in UDSC-EVs by Chen et al. and might improve the anti-apoptotic properties of osteoblasts and endothelial cells, therefore playing a protective function (Chen et al., 2020).

## 5 UDSCs vs*.* U- IPSCs

UDSCs were previously classed as cells with multipotent characteristics, indicating that they may transform into several varieties of cells within the same lineage. Furthermore, they are capable of being turned into induced pluripotent stem cells., which have greater effectiveness (Guan et al., 2014b). However, a new study discovered that empty urine includes many functional epithelial cells that may be well cultivated and transformed into IPSCs (Kibschull et al., 2023).

There are two fundamental methods for creating a significant amount of different cell numbers: the first is cellular reprogramming using UIPSCs, and the second is immediate reconfiguration from baseline UDSCs (Table 5). Cell reprogramming, which is commonly performed through the viral or non-viral administration of reprogramming reagents, transforms UDSCs into proliferative U-IPSCs that can then be transformed into numerous cellular kinds utilizing established procedures. While this approach is quite efficient for creating vast quantities of cells, it is not without risk because of the reprogramming reagents. U-IPSCs may divide into various kinds of cells; U-IPSCs frequently develop into specific cell types by employing specialized induction procedures and triggers that mimic physiological phases of development. Here are some instances of how U-IPSCs can differentiate (Liu et al., 2021).

In preclinical research, developed u-iPSC-derived renal cells were used as an option for regenerative therapies. U-IPSCs have also demonstrated their ability to turn into various cellular lineages. The usefulness and productivity of techniques for development can change according to cellular species and techniques employed (Mulder et al., 2020). Continuing studies are concentrated on improving differentiation strategies to boost the efficiency and usability of U-IPSCs for regenerative therapies and disease models. The direct technique includes changing critical transcriptional genes. It aims to turn UDSCs directly into targeted kinds of cells without crossing via the iPSC stage, possibly preserving lineage-specific data while addressing safety issues. Numerous investigations proved that UDSCs can be directly transformed into multiple cell lines (Liu et al., 2021).

It is essential to stress that direct reprogramming investigations are just starting, and further investigations are essential for properly comprehending the possible uses of this technique for therapeutic purposes.

## 6 Employment of UDSCs in pathological simulation

**FIGURE 3:**
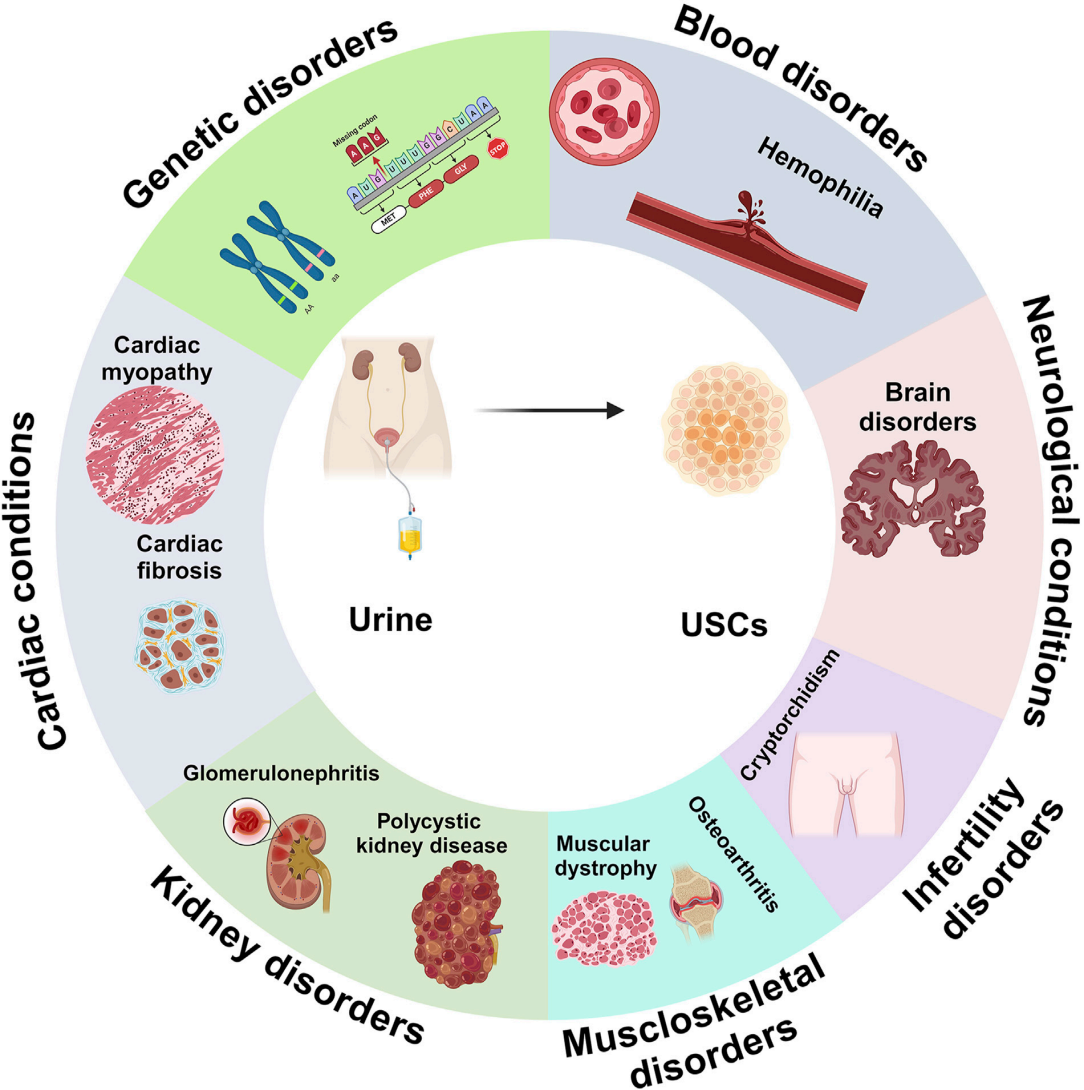
Adaptability of UDSCs as large-scale, ethical approach for disease modelling in numerous diseases and conditions, such as blood disorders, neurological conditions, infertility isorders, musculoskeletal disorders, kidney disorders, cadiac conditions, and genetic disorders.

U-IPSCs have been employed in disease models for researching an extensive spectrum of human illnesses, allowing researchers to understand the molecular processes, pathophysiology, and prospective treatment methods to address diverse ailments (Figure 3) (Li et al., 2017b). Furthermore, U-IPSCs obtained from those with certain genetic conditions can develop into illness-related cellular populations to simulate the condition *in vitro*. This strategy has been utilized to investigate pathological conditions (Li et al., 2017b; Shi and Cheung, 2021).

Investigators can learn more about disease causes, evaluate possible medication candidates, and investigate personalized medicine techniques by examining u-iPSC-derived cells from afflicted patients. U-IPSCs have been employed to imitate several neurological illnesses; neurogenic transformation of U-IPSCs allows researchers to detect illness-specific cellular characteristics, evaluate disease development, and test novel treatments (Shi and Cheung, 2021). U-IPSCs have also been utilized to simulate cardiac illnesses (Huang et al., 2020). Differentiation of U-IPSCs into cardiomyocytes allows scientists to explore disease processes, assess aberrant cardiac cell function, and test prospective therapy techniques (Huang et al., 2020).

Furthermore, U-IPSCs have been used to imitate various nephrological conditions and acute renal damage. The transformation of U-IPSCs into kidney cellular lineages enables scientists to evaluate illness-specific cellular characteristics, track disease development, and test novel therapeutic approaches for kidney ailments (Bharadwaj et al., 2013).

Guo et al. obtained u-iPSC from a 5-year-old boy suffering from X-linked Alport Syndrome (X-LAS) and showed the practicality as a cellular-orchestrated illness model by validating the pluripotency capacity and proper karyotyping (Guo et al., 2020).

Hemophilia is a widespread inherited hemorrhaging disorder (Shah et al., 2023). Because of deficiencies in the way blood clots, those who have severe hemophilia could experience fatal episodes of bleeding during trauma or surgeries (Gualtierotti et al., 2024). The primary therapy is to replace the deficient factors (Koga et al., 2023). Lu et al. produced U-IPSCs from a Hemophilia A patient with an Inv22 mutation (Lu et al., 2020). The pathogenesis of neurological diseases is multifactorial, including intricate links between genetics and the environment that cannot be fully replicated in animal models (Yau et al., 2023).

Teles et al. developed human cerebral organoids from U-IPSCs obtained from Down syndrome (DS) patients, expressing evolutionary patterns of the early-stage forebrain (Teles e Silva et al., 2023). Musculoskeletal illnesses are the second most common cause of disability worldwide, inflicting a considerable cost on society (Giardullo et al., 2021). Autosomal dominant osteopetrosis type II (ADO2), a dominantly inherited musculoskeletal condition, causes fractures, joint discomfort, and bone morphological abnormalities (Lu et al., 2022). Ou et al. generated U-IPSCs from ADO2 patients and detected the identical CLCN7 polymorphism (R286W) in their specimens by juxtaposing them with ADO2-IPSCs (Ou et al., 2019).

Cryptorchidism is a frequent congenital condition in newborns, with the potential for infertility. Because human embryos are difficult to obtain for scientific research, *in vitro* models may be useful (Li et al., 2024). Zhou et al. used lentivirus to reprogram UDSCs from a cryptorchid patient, which co-cultured onto irradiated mouse embryonic fibroblasts and cultivated using human embryonic stem cells (ESCs) media. Their findings showed that these two cell lines resembled human ES cells in terms of morphology, biomarker transcription, and pluripotency-associated genes (Zhou et al., 2013).

## 7 Therapeutic and regenerative applications of UDSCs

**FIGURE 4:**
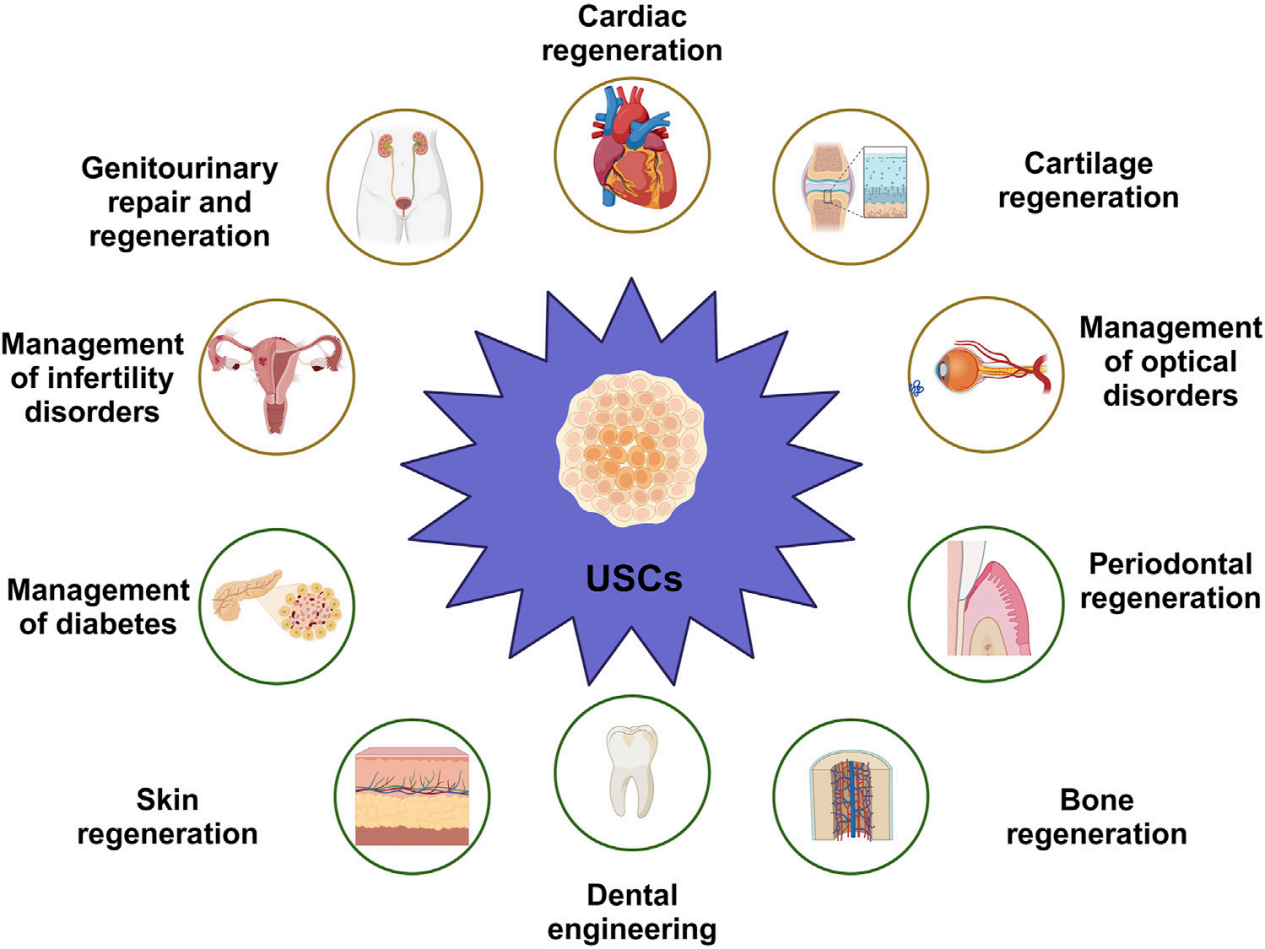
Versatility of UDSCs as economic, non-invasive therapeutic and regenerative approach in numerous applications, such as cartilage regeneration, management of cardiac conditions, management of optical disorders, periodontal regeneration, dental engineering, skin regeneration, management of diabetes, management of infertility disorders in both of males and females, and genitourinary repair and regeneration.

Concerning the previously mentioned characteristics, UDSCs represent a promising option as a source for stem cells (Table 2). With the utilization of proper scaffolding material, the administration of UDSCs could be a promising therapeutic modality in addressing periodontal defects (Yang et al., 2020; Xing et al., 2022) (Figure 4).

**TABLE 2:** UDSCs treatment for illnesses in different tissues and organs.

Application	Procedure	Model	Outcomes	Advantages	Limitations	Recommendation	References
Genitourinary repair and regeneration	UDSCS	*In vitro*: UDSCs *In vivo*: rabbits	*In vitro*: UDSCS differentiated into myogenic and urothelial like cells *In vivo*: promote urethral reconstruction, as demonstrated by substantial increases in urethral diameter, rejuvenation velocity, muscle content, and vascularization	USCs that are effortlessly separated by voided urine may be widely increased with elevated quantities	Molecular mechanisms were not clarified	Additional studies are required to transfer such technology into the clinic	Liu et al. (2017)
Cartilage regeneration	ACM/hUSCs	*In vitro*: hBMSCs and hUDSCs *In vivo*: rabbits	*In vitro*: hUDSCs had a higher potential for division, colony formation, and migration than hBMSCs in the same passage *In vivo*: considerably stimulated cartilage defect healing	hUDSCs can be regarded an alternative to typical stem cells for cartilage repair	A fferent cellular microenvironment provided by the media may affect cellular behaviors Absence of investigation of interindividual differences	Further examined utilizing cell surveillance, genomic assessments, and another research	Sun et al. (2021b)
Skin wound healing	UDSCs-PCL/GT	*In vitro*: UDSCs *In vivo*: Rabbits	*In vitro*: UDSCs may develop into osteoblasts, adipocytes, and chondrocytes. *In vivo*: UDSCs-PCL/GT-treated lesions healed considerably quicker, with enhanced re-epithelialization, collagen production, and angiogenesis	Non-invasive procedure	Failure to regenerate skin appendages	More research on higher animal models is needed	Fu et al. (2014)
Diabetes	UDSCs	*In vitro*: UDSCs and hADSCs	UDSCs can be transformed into insulin-generating cells	Easy and inexpensive isolation procedure	The levelof responsiveness to glucose was lower than in the main islets	Additional research is required	Hwang et al. (2019)
Dental engineering	UDSCs	*In vitro:* UDSCs *In vivo:* mice	*In vitro*: UiPSCs-derived epithelial sheets developed into ameloblasts in dental-like structures *In vivo*: Regenerative teeth comprise enamel with human-derived ameloblast-like cells and have physical qualities similar to conventional human teeth	Noninvasive technique	Un clear molecular processes	Future research ought to concentrate on the derivation of odontogenic capacity of Ifhu-iPSCs	Cai et al. (2013)
Periodontal regeneration	UDSCs/PDLSCs sheets	*In vitro*: PDLSCs *In vivo*: mice	*In vitro*: When combined with UDSCs, PDLSCs could exhibit enhanced division and cementogenic/osteogenic transformation *In vivo*: generated more fresh and dense tissues while also expressing greater quantities of osteogenic and cementogenic proteins	Noninvasive collecting approach to uscs and their steady growth render	Cellular process of this experiment deserves additional investigation	These findings need more investigation point to a potential new technique for clinical periodontal tissue restoration	Yang et al. (2020)
Ophthalmological disorders	UDSCs-derived exosomes	*In vitro*: RGCs	Suppressing cell apoptosis, increasing cellular survival, and promoting the division of aged rgcs	Exosome targeted therapy is a fresh strategy to cell-free therapy and medication delivery for the management of age-related illnesses	Genetic variations as well as modifications in communication signaling need more clarification	In general, attention to the influence of ageing on USCs and *vice versa* is crucial for devising innovative therapy employing USCs with an emphasis on the treatment of older adults	Dan et al. (2023)
Female infertility	SIS loaded with UDSCs	*In vitro:* HEEC and hESC. *In vivo:* Rats	*In vitro*: the SIS scaffolds showed good mechanical characteristics and biocompatibility while promoting epithelial migration and revascularization *In vivo*: Transplantation of SIS/UDSCs preserved standard luminal structure, increased endometrial and glandular rejuvenation, revascularization, reduced fibrosis, and enhanced endometrium responsiveness	This has resulted in a new technique for boosting endometrial shape and functioning, which could prove useful for IUA avoidance and management in clinics	The biological process behind the unique role of the USCs to the endometrial and gland regeneration is inadequately known	It deserves rigorous and comprehensive research	Song et al. (2023)
Male infertility	UDSCs and UDSCs-EXOs	*In vivo*: Mice	Recovered spermatogenesis in NOA mice 36 days following busulfan administration	This investigation offers a unique perspective on the therapy of NOA	Failed to shield the mouse testicles from early busulfan damage	More investigations are needed in higher animal models	Deng et al. (2019)
Cardiac regeneration	UDSCs	*In vitro*: UDSCs *In vivo*: Rats	*In vitro*: cellular architectures were altered to produce endothelial and smooth muscle-like cells *In vivo*, the application of UDSCs could alleviate fibrosis and apoptosis of the myocardium in rats. Injection of USCs restored the poor functioning of the left ventricle	A viable therapy strategy for problems might involve reducing fibrosis and preventing cell death	Unclear mechanism	Additional studies should look at the mechanism and therapeutic applications	Dong et al. (2016)
Bone engineering	Calcium silicate (CS) particulates, and mixed with poly (lactic-co-glycolic acid) (PLGA)/	*In vitro*: UDSCs *In vivo*: Rats	*In vitro*: boosted cell division, ALP action, calcium deposits, and strongly stimulated the bone-forming transformation *In vivo*: Promoted the synthesis of new bones significantly	Non invasive	The precise mechanism behind this phenomenon remains obscure and requires additional exploration	These findings can help guide future research on biomaterial development	Guan et al. (2015a)

ACM, Acellular cartilage extracellular matrix; ALP, Alkaline phosphatase; CS, Calcium silicate; GT, Gelatin; HADSCs, Human adipose stem cells; HEEC, Human endometrial epithelial cell; HESC, Human endometrial stromal cell; Ifhu-iPSCs, Integration-free human urine induced pluripotent stem cells; IUA, Intra- uterine adhesion; NOA, Non obstructive azoospermia; PCL, Polycaprolactone; PDLSCs, Periodontal stem cells; PLGA, Poly (lactic-co-glycolic acid); RGCs, Retinal ganglion cells; SIS, Small intestine submucosa; UDSCS, Urine derived stem cells; UiPSCs, Urine induced pluripotent stem cells.

### 7.1 Genitourinary repair and regeneration

UDSCs, which originate in the urinary tract system, are an excellent alternative for repairing genitourinary organs (Table 3) (Liu et al., 2017). Application UDSCs have shown tremendous potential for treating acute kidney injury (AKI). For example, UDSCs or UDSCs-Exos can reduce cell death, control inflammatory reactions, and enhance renal functionality (Tian et al., 2017). UDSCs-Exos can protect kidney cell function during hypoxia/reoxygenation *in vitro* by transferring functional microRNAs (Zhang et al., 2020c). UDSCs could reduce fibrosis in the kidneys and inflammatory conditions and protect kidneys from damage through paracrine effects in rats (Xiong et al., 2020).

**TABLE 3:** UDSCs in genitourinary repair and regeneration.

Platform	Method of application	Model	Outcomes	References
USCs-Exos		*In vivo*: Rats	Lower tubular damage scoring, more dividing cells, and fewer apoptotic cells	Tian et al. (2017)
	Intravenous	*In vivo*: Rats	Could efficiently reduce apoptosis and provide functional preservation	Zhang et al. (2020c)
	Intra-lesion injection	*In vivo*: Rats	Revealed reduced function loss, cell penetration, and oxidative stress	Xiong et al. (2020)
	Intravenous	*In vitro*: HK2 (human kidney cortex) cells *In vivo*: Rats	*In vitro*: Suppression of nuclear factor (NF)-κB signaling and protecting HK2 cells from damage *In vivo*: Protected kidneys from damage	Li et al. (2020)
	Intravenous	*In vitro* *In vivo*: protamine/lipopolysaccharide (P/LPS)-induced interstitial cystitis in a rodent	UDSCs recovered bladder functionality and histological integrity	Li et al. (2017a)
SIS scaffold/UDSCs	Implantation	*In vitro* *In vivo*: Rabbit	The urethral diameter, urothelial regeneration velocity, smooth muscle content, and vascular density all increased dramatically	Liu et al. (2017)

Exos, Exosomes; LPS, Lipopolysaccharide; P, Protamine.

Bladder regeneration has been viewed as a viable approach to restoring functionality in individuals suffering from severe bladder disorders, including congenital abnormalities and malignancies (Sharma et al., 2013). Intravenous injection of UDSCs dramatically reduced oxidative stress, inflammatory reactions, and cell death in bladder tissues in mice. USCs can be accessible using a straightforward, noninvasive, and low-cost technique that does not need surgical operations. This finding might pave the way for a potential clinical investigation to evaluate the therapeutic effectiveness of USC in treating interstitial cystitis. (Li et al., 2017a).

Urethral defects are often treated with reconstructive surgery; however, this remains a considerable difficulty due to a variety of postoperative consequences (Hua et al., 2023). Because of the shortage of autogenic grafts for urethral repair, there has been much enthusiasm for bioengineered urethras. Given their multipotent capacity, UDSCs offer significant potential for urethral regeneration (Casarin et al., 2022).

UDSCs-loaded SIS scaffolds were found to promote urethral reconstruction, as demonstrated by substantial increases in urethral diameter, rejuvenation velocity, muscle content, and vascularization. There are several restrictions. First, scientists did not examine the bioactive substances produced by USCs that are thought to improve tissue regeneration. These include growth factors and cytokines. Second, in the current study, urethral defect models were produced in normal animals, that can’t adequately imitate the clinical state of urethral strictures, which are distinguished by a fibrotic urethral bed. Finally, just a few test subjects were examined at each time point. Additional studies are required to transfer such technology into the clinic (Liu et al., 2017).

### 7.2 Cartilage regeneration

Due to cartilage’s weak intrinsic healing capabilities, stem cells have sparked interest in articular cartilage repair. Autogenic stem cells are the golden standard in tissue engineering; however, most conventional stem cells need invasive and complex steps to use (Zha et al., 2021). UDSCs are an innovative stem cell reservoir that may be obtained non-invasively and repeatedly from the same person (Table 4).

**TABLE 4:** UDSCs in cartilage regeneration.

Platform	Application	Model	Outcomes	References
HA/UDSCs	IA	Rabbit knee joint with cartilage defect	HUDSCs-HA might trigger much greater neocartilage production by overexpression of aggrecan and collagen II.	Chen et al. (2018)
ACM/hUDSCs or hBMSCs	IA	*In vitro*: hUDSCs hBMSCs and *In vivo*: Rabbits	*In vitro*: HUDSCs demonstrated a higher potential for division, colony formation, and motility than hBMSCs in the same passage *In vivo*: Bioactive scaffolds significantly stimulated cartilage regeneration in the rabbit knee model 12 weeks after placement, with the regenerated tissue primarily consisting of hyaline cartilage	Sun et al. (2022a)
porcine SIS hydrogel hUDSCs	Intro lesional injection	*In vitro*: hUDSCs, and RAW264.7 cells *1* *In vivo*: Rats with tendon-bone interface	*In vitro*: Outstanding biological compatibility, and enhanced macrophage polarization from M1 to M2 *In vivo*: SIS hydrogel loaded with hUDSCs that excessively expressed bFGF in an optimal immunological environment is expected to increase hUDSCs’ chondrogenic capacity, assisting in the functional repair of TBI injuries	Chen et al. (2024)
HUDSC-EXOs)	IA	*In vitro* *In vivo*: Rats	*In vitro*: Chondrocytes fed with IL-1β via hUDSCs-Exos showed higher division and migration, while inhibiting apoptosis. However, ECM secretion reduced *In vivo*, IA injections of hUDSCs-140-Exos improved costochondral regeneration	Liu et al. (2022)
UDSCs-UECM)	IA	*ex vivo:* Rabbits IPFSCs *In vivo:Rabbits*	Enhanced cartilage engineering	Pei et al. (2023)
injectable pig cartilage-derived dECM hydrogels	IA	*In vitro*: UDSCs *In vivo*: Rats	*In vitro*: ECM hydrogels can promote chondrogenic development in UDSCs *In vivo*: Enhanced extracellular matrix production, regulated immune system activity, and stimulate cartilage regeneration	Zeng et al. (2022)

ACM, Acellular cartilage extracellular matrix; BFGF, Basic fibroblast growth factor; DECM, Decellularized extracellular matrix; ECM, Extracellular matrix; HA, Hyaluronic acid; HBMSCs, Human bone marrow stem cells; HUDSC-Exos, Exosomes derived from human urine derived stem cells; HUDSCs, Human urine derived stem cells; IPFSCs, Infrapatellar fat pad-derived stem cells; SIS, Small intestinal submucosa; TBI, Tendon-bone-injury, UDSCs, Urine derived stem cells.

Chen et al. initially verified the potential of UDSCs for cartilage healing in 2018 (Chen et al., 2018). They found that induced hUDSCs produced chondrogenic biomarkers such as aggrecan and collagen II, and their transcript levels were elevated *in vitro*. In addition, they mixed hUDSCs with hyaluronic acid (HA) and administered in joint defect in rabbits. Twelve weeks following treatment could encourage cartilage regeneration. These results indicated that hUSCs might be another potential medicinal cell origin for cartilage tissue engineering, particularly if coupled with HA. Despite the outcomes of this investigation seem intriguing, numerous constraints must be tackled before therapeutic applications, and more research is necessary to demonstrate: 1) how to boost hUSCs’ chondrogenic capability since the expression levels of all chondrogenic genes were comparatively low; 2) the benefits of hUSCs over other MSCs by comparing their chondrogenic ability to that of other MSC types, including hBMSCs and hASCs; and 3) the biological processes that govern the chemical reactions between hUSCs and HA (Chen et al., 2018).

Sun et al. tested the chondrogenic biological activities of hUDSCs and hBMSCs from the same person. *In vitro* tests showed that hUDSCs had a higher potential for division, colony formation, and migration than hBMSCs in the same passage. At 12 weeks after placement, scaffolds loaded with hUDSCs or hBMSCs considerably stimulated cartilage defect healing in the rabbit knee model, with the regenerated tissue primarily being hyaline cartilage. Nevertheless, there was not a substantial distinction in cartilage repair efficacy between hUDSCs and hBMSCs. However, this investigation has certain drawbacks. First, since various media can sustain the steady and fast proliferation of hUSCs and hBMSCs, respectively, the micro-environment supplied by the media may influence cellular activities *in vitro*. Moreover, there are differences in cellular activities across stem cells from various donors. Further investigations should also investigate interindividual differences.

In general, hUSCs can be regarded as an alternative to typical stem cells for cartilage repair; however, differences in the capability of hUSCs and hBMSCs to stimulate cartilage regeneration *in vivo* and *in vitro* should be further examined utilizing cell surveillance, genomic assessments, and other research (Sun et al., 2022a).

The tendon-bone interface (TBI) is critical in the transmission of mechanical stresses, yet it is vulnerable to injuries, which usually contribute to fibrous scar tissue development (Chae et al., 2021). As a result, rejuvenation of the fibrocartilaginous zone has become a critical topic of study. Currently, the utilization of stem cells offers an auspicious method for TBI therapy (Yea et al., 2020).

Small intestine submucosa (SIS) hydrogel loaded with hUDSCs that excessively express bFGF was utilized to treat TBI damage, correct inflammatory response abnormalities, and promote the polarizing of macrophages towards regenerative characteristics, establishing a favorable immunological milieu. The amalgamated impact of essential fibroblast growth factor (bFGF) and an optimal immunological environment are expected to boost hUDSCs’ chondrogenic capacity, assisting in the functional recovery of TBI injuries. There are several drawbacks to this research. First, while macrophages are the subject matter, other immune cells like T and B cells may potentially have a substantial impact on TBI repair, but their functions have not been investigated in this study. Second, the specific processes via which SIS hydrogel modifies macrophage morphologies have yet to be identified. Third, despite the multiplicity of macrophage phenotypes characterized by functioning, this study simplifies them into M1 and M2 categories. As a result, future research must thoroughly examine macrophage-produced growth factors, describe the interaction of diverse inflammatory mediators, and document the temporal-spatial patterns of macrophage phenotypic development during TBI repair. Notwithstanding these limits, previous data indicate that SIS hydrogel, especially when paired with hUSCs-bFGF, is a viable approach for enhancing TBI recovery (Chen et al., 2024).

In an investigation conducted by Liu et al.,, chondrocytes injected with IL-1β via hUDSCs-EXOs showed increased proliferation and migration, while apoptosis was prevented. However, extracellular matrix (ECM) secretion diminished as an undesirable effect. HUDSCs-140-Exos preserved the benefits of hUDSCs-EXOs while further increasing ECM secretion by targeting VEGFA, such as collagen II and aggrecan. *In vivo* testing proved they could promote osteochondral regeneration. While IL-1β activation and surgical initiation are commonly utilized to simulate OA-like changes, the pathophysiology of OA is far more complicated. Second, they employed female rats to imitate *in vivo* OA in their investigation, because estrogen has been shown to boost miR-140 expression, protecting cartilage from degeneration. As a result, they must confirm their findings using male rats (Liu et al., 2022).

As verified by proteomics data, UDSCS decellularized ECM (dECM) improved rabbit IPFSC cartilage reconstruction and functionality restoration, especially for UDSCs-deposited dECM. RNA-Seq research revealed that inflammatory triggering of macrophages and polarization, as well as mesenchymal-epithelial transition (MET), may be implicated in the C-dECM-mediated increase of IPFSCs’ chondrogenic ability, requiring additional investigation (Pei et al., 2023).

Furthermore, Zeng et al. found that UDSCs in dECM hydrogel survived, multiplied, and formed a large amount of cartilaginous ECM comprising collagen II and aggrecan. In addition, This bioactive platform could stimulate extracellular matrix production, regulate the immune system’s reaction, and enhance cartilage repair in rats (Zeng et al., 2022).

### 7.3 Skin wound healing

Healthy skin is critical for sustaining the physiological equilibrium because it defends against infection, loss of minerals, mechanical stresses, and temperature imbalance. It also plays an important role in dynamic processes, including hydration, vitamin D production, and excretion (Kim et al., 2021).

Consequently, any interruption in skin integrity may lead to tissue breakdown, leading to acute or chronic lesions. Acute wounds are injuries caused by injuries such as burns or surgically created wounds that heal in a reasonable amount of time, while chronic wounds like ulcers or post-surgical wounds do not progress via the standard healing process promptly, leading to an absence of substantial rehabilitation over an extended period (Zhong et al., 2022). UDSCs could perform an important part in the promotion of skin wound regeneration (Table 5). For example, UDSCs combined with a nanofibrous membrane composed of polycaprolactone/gelatin (PCL/GT) significantly improved revascularization and wound recuperation in rabbits. These findings also show that adding gelatin significantly enhances the hydrophilicity and mechanical characteristics of PCL/GT fibrous membranes, obtaining values equivalent to those of human skin tissue (Fu et al., 2014).

**TABLE 5:** UDSCs in Skin wound healing.

Platform	Application	Model	Outcomes	References
PCL/GT membranes/UDSCs	Transplantation	*In vitro*: UDSCs *In vivo*: Rabbits	*In vitro*: PCL/GT membrane exhibits mechanical characteristics similar to skin tissue and high biocompatibility UDSCs release VEGF and TGF-β1, and their conditioned media promotes endothelial cells to migrate, divide, and form tubules *In vivo*, UDSCs-PCL/GT-treated wounds healed considerably quicker, with more re-epithelialization, collagen production, and angiogenesis	Fu et al. (2014)
BC		*In vitro*: EA.hy926 *In vivo*: Rats	*In vitro*: Could boost EA.hy926 development and longevity, whereas hUDSCs-CM may promote EA.hy926 multiplication on BC *In vivo*: wound healing rate was dramatically enhanced, with quicker re-epithelialization, collagen synthesis, and neovascularization	Cao et al. (2019)
SIS	Transplantation	*In vitro*: UDSCs *In vivo*: Mice	*In vitro*: UDSCs demonstrated outstanding cell survival and architecture *In vivo*: Dramatically increased the wound healing capability o. It increased wound angiogenesis, encouraged re-epithelialization, and enhanced collagen fiber accumulation and remodeling in the latter stages of wound healing	Zhang et al. (2020b)
BG	Transplantation	*In vitro*: Coculture of UDSCS, endothelial cells and fibroblasts *In vivo*: Mice	*In vitro*: Increased capillary-like network development of endothelial cells, matrix protein synthesis, and myofibroblast transformation of fibroblast *In vivo*: BG-activated UDSCs outperformed untreated UDSCs in wound healing by increasing angiogenesis and the buildup of collagen in wounds	Zhang et al. (2018)

BC, Bacterial cellulose; BFGF, Basic fibroblast growth fator; BG, Bioglass; EGF, Epidermal growth factor; G, Gelatin; PCL, Polycaprolactone; SIS, Small intestine submucosa; UDSCs, Urine derived stem cells

Bacterial cellulose (BC) has good physical and chemical characteristics, and it has a strong influence on wound healing (Liu et al., 2025). Given the superior qualities of bacterial cellulose, the surface structured BC is built using a modified version of the “contact guidance” hypothesis, with the goal of influencing cell activity via the arrangement of the material surface to accomplish what is needed for therapy (Liu et al., 2024; Don et al., 2025). Cao et al. discovered that combining the bacterial cellulose and UDSCs improved revascularization and wound rejuvenation in comparison with treating with scaffold or UDSCs alone (Cao et al., 2019). When activated by bioglass, UDSCs may upregulate growth factor expression, resulting in more favorable wound healing results (Zhang et al., 2018).

### 7.4 Diabetes

Diabetes mellitus type 2, the ninth most significant cause of mortality, causes insulin resistance and loss of pancreatic β-cells (El-Sherbiny et al., 2020). Isolated UDSCs have been shown to develop into insulin-producing β cells and enhance pancreatic islet angiogenesis, making them appropriate for diabetes therapy (Zhao et al., 2018; Hwang et al., 2019). Hwang et al. discovered that UDSCs can be transformed into insulin-generating cells, which makes reestablishing functioning insulin-releasing cells derived from UDSCs preferable to organ transplantation simply because of their relative abundance. Though additional research is required, their findings indicate that hUDSCs might be a promising source for cell treatment in type 1 diabetes (Hwang et al., 2019).

Given that previous research on UDSCs showed better glucose tolerance in diabetic mice, these findings appear inconsistent. Intrapancreatic administrations of UDSCs could represent the most efficient technique, but intravenous administration had an insufficient impact on reducing blood glucose due to the possibility of entrapment in unwanted tissues. Dong et al. found that USCs repaired and protected pancreatic islets in diabetic rats. Therapy with USCs dramatically reduced histological damage and functional deterioration.

While the USC therapy failed to significantly lower fasting blood glucose levels, it did considerably suppress fibrosis and apoptosis. The present research suggested that administering USCs might be beneficial in the treatment of diabetic problems. Regional administration, intra-arterial delivery, or increasing paracrine activity might all be helpful therapy options for wounded tissues. Additional studies should look at the therapeutic benefits of USCs in T2D and its consequences (Dong et al., 2016).

### 7.5 Dental engineering

The tooth is generated by a mutually beneficial interplay between the epithelium and mesenchymal cells (Olaru et al., 2021). The epithelium then divides into ameloblasts, eventually becoming the enamel, whereas the mesenchyme develops into other dental structures. Nevertheless, certain critical difficulties must be addressed before these emerging ideas may be used in dentistry clinics. The paucity of persistent avenues for epithelial stem cells with odontogenic capacity in adult humans is likely the most significant restricting issue (Shen et al., 2020; Lee et al., 2022). In this context, Cai et al. differentiated U-IPSCs into epithelial sheets, which were reorganized with E14.5 mouse dental mesenchyme. Dental-like constructs have been isolated within 3 weeks, with yields of up to 30% for eight distinct iPSC lines, similar to H1 hESC. They also discovered that UiPSC-derived epithelial sheets developed into ameloblasts in dental-like structures, which had physical attributes similar to those observed in regular human teeth, like elastic modulus and hardness.

Of course, further work is needed to generate an iPSC-derived epithelial sheet with odontogenic capability through a deep examination of molecular processes linked with epithelial-mesenchymal reactions, which is a recurring theme throughout development. However, future research ought to concentrate on the derivation of the odontogenic capacity of integration-free human urine induced pluripotent stem cells (ifhU-iPSCs), which will be beneficial for the ultimate objective of the entire regeneration of human teeth for therapeutic therapy (Cai et al., 2013).

### 7.6 Periodontal regeneration

Over the past 150 years, significant scientific discoveries in periodontology have profoundly revolutionized how doctors diagnose and treat periodontal illnesses. Nevertheless, there is still no optimum treatment method today to treat periodontitis or accomplish reliable and efficient tissue regeneration (Karobari et al., 2022; Marya et al., 2022). In many situations, contemporary periodontal therapy fails to regain functionality in various tissues (El-Nablaway et al., 2024b; Atia et al., 2023). Traditional mechanical or anti-infective periodontal therapy has aimed to eliminate inflammatory reactions and slow disease development (Rokaya et al., 2022; Heboyan et al., 2022). In the last 20 years, many regeneration techniques have been devised, tried, and reviewed to repair missing tooth-supporting components (Banakar et al., 2022; Moonla et al., 2022).

Human periodontal ligament stem cells (PDLSCs) have been used extensively as seeding cells and cellular sheets in periodontal tissue regeneration (Atia et al., 2025). Despite substantial advances in PDLSC application, promoting cell proliferation and numerous differentiations of PDLSCs remains a key problem due to low cell numbers at the time of acquiring (Alkandari et al., 2025; El-Nablaway et al., 2024a). Yang et al. investigated the paracrine impacts of UDSCs on cell division and bone-forming transformation in PDLSCs. When combined with UDSCs, PDLSCs could exhibit enhanced division and cementogenic/osteogenic transformation. *In vivo* implantation revealed that PDLSC sheets cocultured with UDSCs generated more fresh and dense tissues while also expressing greater quantities of osteogenic and cementogenic proteins.

These findings point to a potential new technique for clinical periodontal tissue restoration. While the cellular process of this experiment deserves additional investigation, the noninvasive collecting approach to USCs and their steady growth render USCs a potential novel method for utilization in clinical periodontal tissue restoration (Yang et al., 2020). Furthermore, Xiong et al. investigated the biological impacts of the hUSC-derived ECM on the division, attachment, dispersing, and transformation of hPDLSCs. They discovered that UDSCs-derived ECM (UECM) increased PDLSC division, osteogenic transformation capacity, and angiogenesis in comparison with native PDLSCs. The drawbacks of this study stem from the inability to precisely quantify the protein content of the ECM. Equivalent quantities of cells underwent stimulation for 8 days to form ECMs.

Yet, the ultimate amount of ECM was challenging to determine since the process of producing ECM is influenced by a variety of circumstances. With the features listed above, UECM might be a potential agent for utilization in 3D bioink applications or biological constructs. Nevertheless, other biological experiments to identify the biological basis of the interaction between ECM and hPDLSCs are deserving of further investigation (Xiong et al., 2019).

### 7.7 Ophthalmological disorders

Retinal ganglion cells (RGCs) are neurons in the retina’s last segment. Their axons are dispersed on the surface of the omentum and gathered in the papilla of the optic tract (optic nerve) (Goetz et al., 2022). UDSCs-derived exosomes have therapeutic value by suppressing cell apoptosis, increasing cellular survival, and promoting the division of aged RGCs. The foundational process consists of various genetic variations as well as modifications in communication signaling. Exosome-targeted therapy is a fresh strategy for cell-free therapy and medication delivery for the management of age-related illnesses.

Despite exosome treatment being a novel method for combating aging, translating preclinical outcomes to the clinic requires more detailed research into exosome biology and associated approaches. In general, attention to the influence of aging on USCs and *vice versa* is crucial for devising innovative therapy employing USCs with an emphasis on the treatment of older adults (Dan et al., 2023).

### 7.8 Infertility disorders

Infertility has developed as a global health concern that can be caused by male, female, or both (Volarevic et al., 2014; Vander Borght and Wyns, 2018). Intrauterine adhesion (IUA), an important factor of recurrent abortions and secondary infertility, is defined as partial or complete obliteration of the uterus owing to injuries to the endometrium. While many therapies have been explored to prevent IUA, they have demonstrated poor therapeutic efficacy. Tissue engineering technological innovation, a promising bioengineering tool, can potentially improve endometrial regeneration.

Small intestine submucosa (SIS) loaded with UDSCs has been used to regenerate endometrial. *In vitro*, the SIS scaffolds showed good mechanical characteristics and biocompatibility while promoting epithelial migration and revascularization. Transplantation of SIS/UDSCs preserved standard luminal structure, increased endometrial and glandular rejuvenation, revascularization, reduced fibrosis, and enhanced endometrium responsiveness. This has resulted in a new technique for boosting endometrial shape and functioning, which could prove useful for IUA avoidance and management in clinics. The biological process behind the unique role of the USCs in endometrial and gland regeneration is inadequately known, although it deserves rigorous and comprehensive research (Song et al., 2023).

Nonobstructive azoospermia (NOA) is a severe condition of male infertility with few viable treatment options (Zhankina et al., 2021). UDSCs have multipotent differentiation potential and paracrine actions and are involved in tissue reconstruction and rejuvenation. Deng et al. demonstrated that both UDSCs and UDSCs-EXOs recovered spermatogenesis in NOA mice 36 days following busulfan administration, but they failed to shield the mouse testicles from early busulfan damage. This investigation offers a unique perspective on the therapy of NOA (Deng et al., 2019).

### 7.9 Cardiac regeneration

Cardiomyocyte loss is connected with a reduction in the heart’s pumping ability. Since cardiomyocytes have an exceedingly poor self-renewal capability (Bergmann et al., 2015). Human genetically acquired heart disorders have been researched regardless of patient genetic origin. Because human cardiomyocytes (CMs) are in short supply, using urine specimens to make U-IPSCs-derived CMs would be a noninvasive way to uncover cardiac disorders that cause diseases in individuals with specific genetic backgrounds.

Jouni et al. modified cells from the urine of a patient with long QT syndrome who had the HERG A561P gene mutation utilizing an episomal-based approach.

UhiPS cells were subsequently differentiated into CMs by the matrix sandwich technique. UhiPS-CMs properly expressed atrial and ventricular myofilament proteins, as well as ion channels. These results suggest the application of CMs derived from iPS cells rather than noncardiac heterologous transcription methods to gain a true understanding of the mechanism behind cardiac channelopathy. Currently, they are the most analogous model to human CMs derived directly from heart tissue, enabling exact knowledge of the molecular foundation that underlies disease phenotypes (Jouni et al., 2015).

Ventricular septal defects (VSDs) are among the most prevalent congenital cardiac abnormalities. Cao et al. used a Sendai virus vector for successfully generating integration-free iPSCs from urine specimens of a patient with VSD and HF who carried an L2483R mutation in the RyR2 gene. U-IPSCs exhibited characteristics of cardiomyocytes, such as spontaneously induced contractions and high levels of cardiac biomarkers. Nevertheless, as compared to cardiomyocytes produced from H9 cells, UDSCs showed a more significant amount of autophagy, indicating that autophagy may play a crucial part in the emergence of VSD with heart failure (HF). The approach reported here enables the affordable and repeatable production of human cardiomyocytes in entirely chemically controlled circumstances, facilitating the implementation of iPSCs into large-scale testing applications or regenerative therapies (Cao et al., 2018). *In vivo,* the application of UDSCs could alleviate fibrosis and apoptosis of the myocardium in rats. Injection of USCs restored the poor functioning of the left ventricle. A viable therapy strategy for problems might involve reducing fibrosis and preventing cell death. Additional studies should look at the mechanism and therapeutic applications (Dong et al., 2016).

### 7.10 Bone engineering

Bone regeneration is important in orthopedic and dental therapy, particularly in traumatic and congenital abnormalities and bone strengthening for biomedical implants. Regardless of the wide range of procedures used in healthcare settings, efficient and functional bone regeneration represents a substantial issue (Gu et al., 2022).

While autogenic bone-based surgeries are regarded as the most effective approach, their practical application remains restricted because of comorbidity at the donation site and a paucity of appropriate bone volume. As a result, these constraints have prompted researchers to investigate artificial substitutes as potential scaffolds or alternatives to bone transplantation (He et al., 2022).

Numerous materials have been created and assessed for their suitability for bone regeneration. Additionally, artificial implantable biomaterials designed to replace natural bone should have bone-like architecture and mechanical characteristics for maximum performance. Stem cell transplantation, which uses the body’s regenerative capacities, offers a cutting-edge way to modify conventional therapies and potentially give more individualized and successful therapies for bone defects. MSCs also produce bioactive chemicals that can modulate immunity system activity and stimulate tissue renewal. As a result, MSCs are widely recognized as a promising tissue regeneration tool (Lau et al., 2022). Autogenous MSCs have shown inadequate efficacy in addressing osteoporosis and bone abnormalities due to diminished functionality and reduced regeneration capacity (Feroz and Dias, 2021; Liu et al., 2020c). Numerous materials have been created and assessed for their suitability for bone regeneration. Additionally, artificial implantable biomaterials designed to replace natural bone should have bone-like architecture and mechanical characteristics for maximum performance. Stem cell transplantation, which uses the body’s regenerative capacities, offers a cutting-edge way to modify conventional therapies and potentially give more individualized and successful therapies for bone defects. MSCs also produce bioactive chemicals that modulate immunity system activity and stimulate tissue renewal. As a result, MSCs are widely recognized as a promising tissue regeneration tool (Anderson et al., 2022).

Bone substitutes could be utilized as an efficient bone regeneration method. A substantial human supply of autologous cells that can develop or be transformed into osteoblasts is vital for designing human bone transplants (Amini et al., 2012). Nevertheless, extensive bone injury necessitates the supply of osteogenic cells as a 3D platform to facilitate bone repair. ESCs and IPSCs are now employed for bone transplants (Guan et al., 2015b).

Furthermore, because of UDSC’s excellent proliferative, self-regeneration, and transformation potential when transformed into osteoblasts, urine stem cells might be an excellent alternative for bone regeneration. Guan et al. used Calcium silicate (CS) particulates and mixed with poly (lactic-co-glycolic acid) (PLGA), to generate PLGA/CS composite. UDSCs were then sown onto these frameworks, and they were then transplanted into naked mice. The findings demonstrated that CS ion extracts boosted cell division, ALP action, calcium deposits, and the synthesis of new bones significantly and strongly stimulated the bone-forming transformation of UDSCs *in vivo.* As a result, PLGA +10% CS scaffolds may have the optimal aforementioned features for USCs. These findings can help guide future research on biomaterial development. Nevertheless, the precise mechanism behind this phenomenon remains obscure and requires additional exploration (Guan et al., 2015a).

## 8 Limitations and concerns

As previously mentioned, gathering urine before the UDSCs separation is straightforward, reliable., innocuous, and inexpensive in comparison to surgical procedures used to collect other stem cell types. UDSCs can be extracted from healthy and diseased people, preserving their ability to grow and divide and allowing for large-scale benign sampling and storage. Moreover, there are no substantial ethical issues related to UDSCs collection, and they can be used for both customized and substantial scientific or therapeutic purposes (Ferreiro et al., 2024).

These changes can be attributed to transforming culturing circumstances, particularly the chemical composition of the medium used for cultivation, or to a natural property of the separated cells that may differ among individuals. Additional investigations are necessary to understand further surface marker levels of transcription and their importance in UDSC development (Wang et al., 2024).

Several essential biological concerns, including immunoregulatory actions and carcinogenic potential hazards, have not been thoroughly investigated to gain additional insight into the usefulness and safety of UDSCs. UDSCs’ immune-modulating impacts must be explored because they are most likely significant (Rao et al., 2024). To produce proof for UDSCs-based therapy, the distinctive characteristics of UDSCs subpopulations must be identified. Furthermore, a fundamental study on the origin of UDSCs is important since it may answer questions including who is best suited to provide UDSCs, the way to collect UDSCs appropriately, and if there are any unique indicators of UDSCs. We hope that single-cell sequencing paired with lineage tracking can provide a few clues.

Clinical-grade stem cell synthesis should adhere strictly to quality assurance (QA) criteria. The reliability and efficacy of the UDSCs must be evaluated (Zhao et al., 2022). Generally, producing UDSCs includes donor selection, cell harvesting, medium composition, cell amplifying process, inspection for quality standards, and so on. First, donor factors, including age, disease, and medications, could impact the biological properties of the UDSCs collected. Gao et al. discovered that UDSCs from younger donors had greater proliferating capacity, less death, and more excellent osteogenic differentiation capability despite UDSCs of all ages having the capacity for bone regeneration (Pizzuti et al., 2024).

Schosserer et al. discovered a more significant successful percentage separating UDSCs from males than from females (70% vs. 42%) (Schosserer et al., 2015). Given the differences between male and female sexual organs, caution should be exercised when gathering specimens of urine from females to avoid the menstrual period and the initial urination of the day, as well as washing the genitalia. Second, the manner of cell collection could impact the final treatment effect; thus it is critical to optimize the UDSCs culture process (Liu et al., 2021; Kibschull et al., 2023).

Third, because medium composition differs by laboratory, the variances are primarily associated with serum levels and nutritional factors. Clinical uses typically demand many cells; thus, the rapid and widespread multiplication of UDSCs for widespread use has presented a significant obstacle. Microcarrier-based suspension cultivation could offer a solution (Hao et al., 2023).

Nevertheless, it is unclear if UDSCs cultivated using this approach stay the same or if the changes between UDSCs cultivated using numerous ways result in various medicinal properties. An alternative way to solve the same challenge is to reconstitute the settings for the cultivation of the UDSCs by including certain nutrients, implanting the UDSCs on frameworks, and controlling oxygen levels to enhance UDSCs separation and division (Kibschull et al., 2023; Liu et al., 2021).

Meeting the high-quality assurance standards is a precondition for UDSCs-based cyto-therapeutics and may require significant efforts. An assay matrix is proposed that includes RNA identification of specific genes, transcriptional evaluation of essential cell surface indicators, and secretome protein identification (Dayati et al., 2024). When using UDSCs in the field of regenerative therapy, the scaffold materials should be evaluated alongside the cellular-scaffolding complexes based on the clinical requirements and the methods via which stem cells can perform biological tasks (Yang et al., 2020; Lu et al., 2023).

Indeed, it is unclear how UDSCs might contribute to tissue restoration. As a result, whether cellular stimulation or other modifications are required remains unclear (Yang et al., 2018). Moreover, the use of UDSCs discharges, including exosomes and ECM, may avoid the possible risks associated with employing UDSCs to treat the condition. It will require an extended period to develop sufficient cells for autologous treatment. As a result, it is greatly practicable to use UDSCs for managing chronic wounds and surgical operations rather than acute burns until allogeneic UDSCs treatments are shown to be safe, successful, and cost-efficient (Yang et al., 2024).

Although U-IPSCs show tremendous prospects for personalized healthcare, various obstacles, and issues should be tackled to realize their capacity completely. IPSCs have significant pluripotency, but their reconfiguration responsiveness varies based on the cellular source, as do their growth courses and development characteristics (Mulder et al., 2020). Furthermore, changes in reprogramming component quantities, mechanisms of transfections, cell cultivation circumstances, and timings among numerous research settings may result in decreased iPSC initiation efficacy and even the creation of off-target cells (Al Abbar et al., 2020). As a result, developing standardized reprogramming techniques is critical to ensuring repeatability and comparability of experiments among various investigation groups, which is essential for enhancing research reliability outcomes. Standardization approaches can involve using a single cell origin, adopting an array of commonly used classical reprogramming factors, ensuring uniformity in experimental circumstances, and developing consistent iPSC recognition standards. Although IPSCs can proliferate indefinitely, the mutation rates vary amongst cell lines. Specific genetic alterations could have been generated during IPSCs processing, resulting in tumorigenesis (De Masi et al., 2020). As a result, preserving genetic integrity in IPSCs throughout long-term proliferation is critical for their safety. To remove potential variances, researchers evaluate the differentiation status, sequence the IPSCs, and analyze the karyotypes. Other options involve employing non-integrative reprogramming strategies, applying genetic modification approaches to fix putative tumor-causing abnormalities in IPSCs, and Pre-differentiation of IPSCs into particular types of cells (Poetsch et al., 2022). To summarize, IPSCs must undergo severe quality assurance and security precautions before being used in therapy to eradicate their cancer-causing capacity.

In several contemporary findings from experiments, IPSCs display standard indicators and have distinct morphological characteristics. Nevertheless, they may not operate properly *in vivo* (Du et al., 2015). Nevertheless, IPSCs can abnormally develop into teratomas, resulting in immunological rejection. Furthermore, longevity and transplantation of IPSCs *in vivo* necessitate adequate criteria, and straightforward cellular injections might not offer the necessary milieu that encourages their differentiation and maturity (Kim et al., 2024). As a result, researchers can examine a variety of approaches, such as selecting the appropriate treatment schedule, sufficient quantities of cells, and employing biomaterials as scaffolding for IPSCs.

After overcoming several laboratory difficulties, the objective is to establish massive iPSC generation to satisfy clinical requirements. First, the choice of a suitable cell origin, like UDSCs, which can be acquired in enormous amounts non-invasively, is critical for wide-scale development (Citterio et al., 2020). Non-integrating reconfiguration approaches for editing genes and optimized Cellular development techniques and the construction of adaptable, computerized culture platforms must be used to increase cell cultivation effectiveness. Furthermore, periodic examination and evaluation of IPSCs, early excision of aberrant cells, and cell quality assurance are required (Citterio et al., 2020). Moreover, reliable filtration and the acquisition of the intended cell types, as well as the construction of proper storage techniques, are required for IPSCs to be used quickly on demand (Jamal et al., 2021).

Although various cell culture settings have been documented, whether these variables have a substantial influence on the medicinal value of USCs is mainly unclear (Xie et al., 2023). The absence of criteria for the classification of UDSCs, in particular, makes direct comparisons between research difficult, which should be tackled in the future. Along with freshly discharged urine, samples for UDSCs separation can be acquired by ureteral catheterization and kept in various conditions (Wu et al., 2011). In addition to healthy donors, USCs from patients with various disorders (e.g., bladder cancer) have been defined (Ooki et al., 2018; Jiang et al., 2016; Zhou et al., 2012). In initial cultivation, UDSCs revealed a rice grain or spindle-like architecture (Chen et al., 2017). Due to long telomeres, USCs can multiply extensively, even after 60–70 cell doublings (Garbe et al., 2009). From a therapeutic standpoint, it is crucial to evaluate the variations in USC biology throughout serial cultures, which is a precondition for determining optimal cell passages for treatment (Sun et al., 2021b; Liu et al., 2022). Donor age, sex, and specimen harvesting techniques also affect the separation and development of UDSCs (Gao et al., 2017). In comparison with older donors, young people demonstrated better functioning, more significant division, and reduced aging in their UDSCs (Ferreiro et al., 2024; Strässler et al., 2018). UDSCs separation was more efficient in male volunteers than with female ones (Klapper-Goldstein et al., 2022; Gutiérrez-Aguirre et al., 2022). Catheterization-collected urine contained more UDSCs than freely voided urine, possibly due to the extraction of cells from the inner bladder wall (Coelho et al., 2023; Yu et al., 2021).

Recommendations on the methodology of urine collection, such as undesirable conditions (e.g., contaminations and cancers) to be utilized in regenerative therapies, have already been published; however, the coming years will bring developments in the automated retrieval of cells from the urine.

## 9 Concluding remarks

UDSCs-dependent periodontal tissue engineering has shown promise in creating a milieu favorable to periodontal regeneration via well-constructed controlled scaffolding constructions that mimic periodontal multilayer frameworks. However, the application of UDSCs continues to encounter various obstacles, notably risks associated with the proliferation of cells *in vitro*, standardized manufacturing processes for biological substances, intrinsic antigenicity, selectivity of scaffold components, biological compatibility, and material breakdown. Cell-free therapy, which incorporates biological compounds to stimulate regeneration in organisms, focused on a number of the drawbacks of cell therapy and provided a better understanding of how UDSCs work. Nevertheless, the setting and conditions of exosome cultivation significantly impact exosome attributes. Further research is needed to define exosome activity, dosage, effectiveness, and appropriate collection and preservation techniques. Finally, pharmacological therapy that targets signaling molecules promotes functioning periodontal regeneration.
